# Does the Component Processes Task Assess Text-Based Inferences Important for Reading Comprehension? A Path Analysis in Primary School Children

**DOI:** 10.3389/fpsyg.2016.00895

**Published:** 2016-06-14

**Authors:** Stephanie I. Wassenburg, Björn B. de Koning, Meinou H. de Vries, Menno van der Schoot

**Affiliations:** ^1^Department of Educational Neuroscience and LEARN! Research Institute for Learning and Education, VU University AmsterdamAmsterdam, Netherlands; ^2^Department of Psychology, Education, and Child Studies, Erasmus School of Behavioral and Social Sciences, Erasmus University RotterdamRotterdam, Netherlands

**Keywords:** component processes task, text-based inferences, reading comprehension, assessment, children, working memory

## Abstract

Using a component processes task (CPT) that differentiates between higher-level cognitive processes of reading comprehension provides important advantages over commonly used general reading comprehension assessments. The present study contributes to further development of the CPT by evaluating the relative contributions of its components (text memory, text inferencing, and knowledge integration) and working memory to general reading comprehension within a single study using path analyses. Participants were 173 third- and fourth-grade children. As hypothesized, knowledge integration was the only component of the CPT that directly contributed to reading comprehension, indicating that the text-inferencing component did not assess inferential processes related to reading comprehension. Working memory was a significant predictor of reading comprehension over and above the component processes. Future research should focus on finding ways to ensure that the text-inferencing component taps into processes important for reading comprehension.

## Introduction

In recent years there has been an expanded interest in the development of a reading-based component processes task (CPT^1^; Hannon and Daneman, 2001; Hannon and Frias, 2012). The purpose of this task is to differentiate between the higher-level cognitive processes of reading comprehension. This way, the CPT contributes to the increasing need to address individual differences in reading performance (August et al., 2006). Moreover, the CPT offers certain advantages over global standardized measures of reading comprehension in that it is theoretically motivated and practical to administer (Hannon and Daneman, 2001).

The idea underlying the CPT is based on a paradigm that was originally developed by Potts and Peterson (1985) to study incorporation of newly learned information into the readers' world knowledge. In the Potts and Peterson paradigm, readers study a three-sentence paragraph in which transitive relations among two real and three artificial terms are described, for example, “A JAL is larger than a TOC. A TOC is larger than a PONY. A BEAVER is larger than a CAZ.” (Potts and Peterson, 1985). The described relations among the items (based on their relative sizes) represent a linear ordering (e.g., JAL > TOC > PONY > BEAVER > CAZ). Importantly, to construct this ordering the reader is required to access and integrate both text-based information (e.g., “A TOC is larger than a PONY”) and prior knowledge (e.g., “a pony is larger than a beaver”), as not all relations are stated explicitly. True-false test statements are used to differentiate between underlying component processes. Text-memory (TM) statements can be answered on the basis of explicitly presented text information (e.g., “A JAL is larger than a TOC”). Text-inferencing (TI) statements test information that can be deduced from the text (e.g., “A JAL is larger than a PONY” can be deduced from the statements “A JAL is larger than a TOC” and “A TOC is larger than a PONY”). Both types of text-based statements do not require prior knowledge. This is left to knowledge-integration (KI) statements which test information that can only be deduced by using existing knowledge in conjunction with presented text information (e.g., “A TOC is larger than a BEAVER” can be deduced from combining the presented text information “A TOC is larger than a PONY” with existing knowledge that ponies are larger than beavers).

Researchers have confirmed, using correlational analyses, factor analyses, and structural equation modeling, that the different types of statements tap into two different resources as theorized: text-based information and prior knowledge (Potts and Peterson, 1985; Hannon and Daneman, 2001; Hannon, 2012; Hannon and Frias, 2012). In addition, research has shown that the KI component predicts readers' ability to construct bridging inferences (Singer and Ritchot, 1996), which is an important skill for reading comprehension. According to Hannon and Daneman (2001), not only the KI component but all components as measured by the paradigm capture aspects of reading comprehension ability. Building on Hannon and Daneman's (2001) attempt to develop an assessment of higher-level component processes of reading comprehension, the Potts and Peterson paradigm has become a popular basis for other CPT's (e.g., August et al., 2006; Hannon and Frias, 2012). In this article, we argue that although there is evidence that the CPT differentiates between different cognitive resources and processes, at least some of these processes do not seem to require comprehension-related activities, and hence not necessarily contribute to reading comprehension. This poses a serious challenge to the CPT presenting itself as an assessment developed to differentiate between multiple components that are all sources of individual differences in reading *comprehension* (Hannon and Daneman, 2001). Our concern focuses on the assumed role of inference in the CPT.

A considerable amount of both developmental and adult research literature has shown that the ability to make inferences is one of the main sources of individual differences in reading comprehension performance (e.g., Oakhill, 1982; Long et al., 1994; Barnes et al., 1996; Cain et al., 2004a). It has been proposed that integrating incoming and previously encountered text information (i.e., text-based inferences) and integrating text information with prior knowledge (i.e., knowledge-based inferences) contribute to the construction of a rich coherent non-linguistic mental representation required for deep-level text comprehension—a so-called situation model (e.g., Zwaan et al., 1995; Zwaan and Radvansky, 1998; McNamara and Magliano, 2009). We contend, however, that the activity of inferential processing related to the construction of a situation model, is not necessarily reflected by the CPT's test statements. That is, the TI component seems to involve transitive inferential processes that are not directly related to reading comprehension, reflecting cognitive skills like logical reasoning instead (i.e., A > B, B > C, so A > C). This may confound the interpretations based on CPT results regarding the reader's actual reading comprehension performance. Arguably, a five-term linear ordering constructed from the text is a linguistic, mathematical mental representation, rather than a rich perceptual mental representation of the described situation. Accordingly, it can be argued that only the KI component entails at least some comprehension-related inferential processing, because it requires readers to move beyond the text and supplement their mental representation with prior perceptual experiences of familiar items.

Support for our argument comes from research administering the CPT in conjunction with general reading comprehension tests. For example, the component scores of the original Potts and Peterson task (Potts and Peterson, 1985) were only moderately correlated to general reading comprehension, whereas they were strongly correlated with deductive and analytical reasoning skill (Hannon and Daneman, 2001; Exp 1). Nearly 60% of the participants reported answering the statements by memorizing and rehearsing a simple linguistic mnemonic for the five-term linear ordering (e.g., JTPBC for JAL > TOC > PONY > BEAVER > CAZ). Further, Hannon and Daneman (2001; Experiment 1) argued that the original CPT was not complex enough to capture the processes that are crucial for reading comprehension. After increasing the complexity of the task (by including more semantic features and increasing the number of test statements; Experiment 2), they indeed showed a stronger correlation to reading comprehension (however yielding comparable correlations to analytical reasoning) and a larger amount of explained variance. Although a difficult task is more likely to tap into complex cognitive processes, this does not necessarily imply that actually comprehension-related reading processes are captured. The five-term orderings constructed from the passages can, presumably, still be memorized and rehearsed as a linguistic mental representation (e.g., a mnemonic) rather than a perceptually rich non-linguistic mental representation. It then is not surprising that for the complex version, the KI component remained to be the best predictor of reading comprehension performance (Hannon and Daneman, 2001).

Further support for our argument comes from the fact that the TI component does not seem to account for any unique variance in general reading comprehension performance over and above TM (Hannon and Daneman, 2001; Hannon and Frias, 2012). This suggests that not all components as assessed by the CPT are sources of individual differences in reading comprehension. Hannon's cognitive components-resource model (2012) shows that the CPT's text-based components together (i.e., TM and TI) have an indirect effect on reading comprehension through the KI component. This model, however, does not differentiate between the effects of TM and TI. Based on results from earlier studies (Hannon and Daneman, 2001; Hannon and Frias, 2012), we hypothesize that this indirect effect is caused by the TM component alone, and that the TI component does not account for unique proportions of variance of reading comprehension. If this is the case, one could wonder to what extent the TI component provides an added value in the CPT.

**Figure 1:**
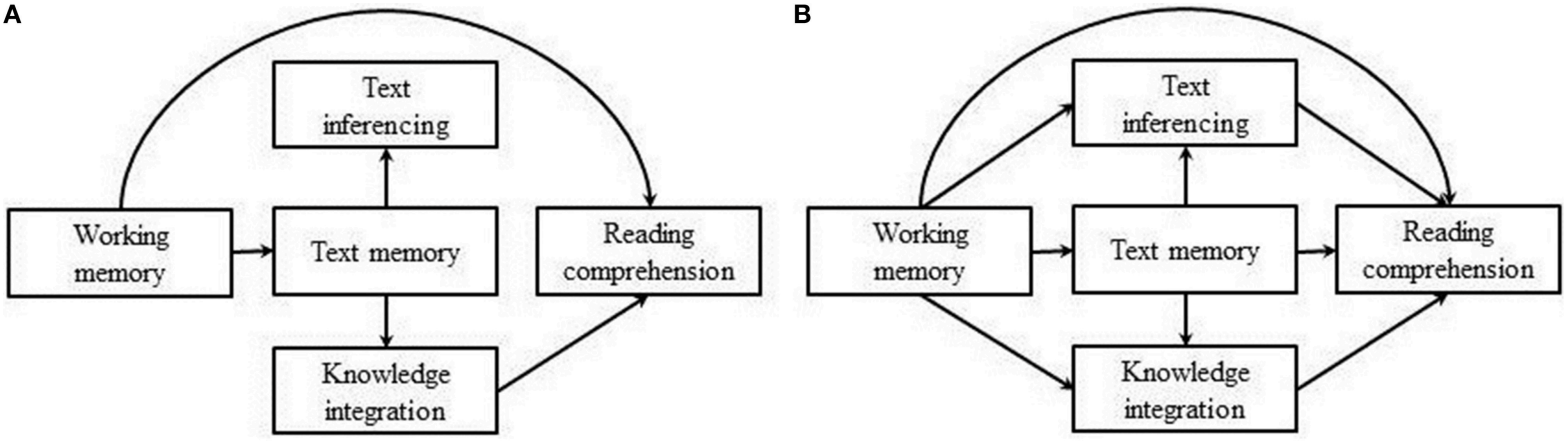
**(A)** Hypothesized model. **(B)** Model 2, including all direct and indirect pathways.

To investigate the concerns described above, a path model (depicted in Figure 1A) showing the hypothesized relative contributions of the CPT components and working memory to reading comprehension performance was tested. The critical aspect of this model is that, consistent with our reasoning, it does not contain a direct path from TI to reading comprehension. Only a direct path from KI to reading comprehension is expected. Working memory (WM) was included in this model, because numerous studies have indicated the crucial role of WM in coordinating construction and integration processes during reading and storage of intermediate and final representations (e.g., Just and Carpenter, 1992; Gathercole and Baddeley, 1993; Ericsson and Kintsch, 1995; Cain, 2006), and its relation to different reading comprehension skills (e.g., Seigneuric et al., 2000). Essentially, comprehension depends on the reader's process of building meaning by actively using knowledge that guides his or her strategies toward this goal (García-Madruga et al., 2013). The relation between WM and reading comprehension has been well established in the literature (see, for example, Daneman and Merikle, 1996), and it has been shown to explain unique variance over and above other traditional predictors, such as word reading, vocabulary, and phonological awareness (Swanson and Howell, 2001; Cain et al., 2004b; Vukovic and Siegel, 2006; Baddeley, 2007). Based on both Hannon's (2012) findings and the literature described above, a direct path from working memory to reading comprehension was included in the presented model. A direct path from working memory to TM reflects the importance of storing information and keeping it active during reading. Only indirect paths, however, from working memory to TI and KI through TM, are included, indicating that readers depend on their memory for literal text statements for correctly answering TI and KI statements, instead of other important meaning-based construction processes in which WM plays an important role. The hypothesized model was tested against a model including all direct and indirect effects (Figure 1B).

Finally, it could be argued that the TI component in fact does assess inferencing skills important for reading comprehension, but this does not surface simply because readers are not required to comprehend the paragraph at a deeper level. To exclude this alternative explanation, the three statements containing crucial information for constructing the five-term ordering were integrated into longer narrative-like texts similar to how this is done in the Diagnostic Assessment of Reading Comprehension (DARC; August et al., 2006). These texts required the reader to make elaborative inferences for deep-level comprehension (for an example see Appendix in Supplementary Material). Practically, this makes the task more ecologically valid as it more closely resembles the regular reading experience. As a result, if the hypothesized path model appears to be a good fit of the data, it can be concluded that the TI component assesses a cognitive skill that does not contribute to deep-level comprehension and, presumably, TI statements are answered based on other types of processing instead, such as relying on logical reasoning skills. Additionally, the present study extends previous research in two ways. First, building further upon Hannon (2012), the present study is—to our knowledge—the first to investigate the relative contributions of three CPT components (i.e., TM, TI, and KI) individually to reading comprehension within a single model. Second, the present study applies the CPT to a yet unexplored target group. Rather than focusing on populations such as adults (Hannon and Daneman, 2001), English language learners (August et al., 2006; Francis et al., 2006), and preschoolers (Hannon and Frias, 2012), our study is the first to investigate the relative contributions of the CPT components and working memory to general reading comprehension performance in primary school children; an age group which represents the ultimate target population for the CPT given the importance of reading comprehension at a primary school level (National Reading Panel, 2000).

## Method

### Participants

The study included data from 173 children (92 boys) in Grades 3 and 4 (age range 8–10 years, *M* = 9.08, *SD* = 0.67) from four regular primary schools in different areas of average to high socio-economic status in the Netherlands. All children were fluent Dutch speakers and came from schools with relatively high concentrations of native Dutch students. Children with (diagnosed) dyslexia or other learning disabilities as indicated by school records (*n* = 17) were excluded from the study. Furthermore, there were no registered cases of children with low IQ (i.e., scores below 85). All participants had grade-appropriate decoding skills as measured by the Een Minuut Toets (EMT; One Minute Test), a standardized Dutch word reading fluency test (Brus and Voeten, 1999).

## Materials

### Component processes task

Our CPT, used to assess the component processes, is a modified version of the DARC (August et al., 2006). The DARC was specifically designed to minimize decoding and language demands and is therefore a suitable task for primary school children. For the present study, the task was slightly modified. Firstly, it was translated in Dutch using only words that would be familiar to children in Grades 3 and 4 (as indicated by their school teachers). Secondly, the names of the artificial terms (i.e., pseudowords) were changed so that they were orthographically transparent and had no meaningful lexical neighbors.

The CPT used in the present study consisted of four short passages. In each passage relations among two real and three artificial terms were described from which a five-term linear ordering could be constructed by using both text information and world knowledge (e.g., MIPPER < BICYCLE < CAR < PLORT < VASKER). Additionally, sentences describing relations among the five terms were incorporated into a narrative depicting a particular story event, including multiple narrative dimensions (i.e., character, time, space, causation) to encourage children to construct a rich mental representation (see the Appendix in Supplementary Material for a story example). To establish and maintain coherence, children were required to engage in inferential processes. Each narrative was followed by 12 true-false statements of three different types; four text-memory statements, three text-inferencing statements, and four knowledge-integration statements (see Appendix in Supplementary Material). To reduce the probability of a correct response through guessing, “I don't know” answer options were added for each statement. The “I don't know” responses were treated as incorrect. Accuracy scores were calculated for the three statement types.

### Working memory

A reading span test (Daneman and Carpenter, 1980) was administered as a measure of verbal working memory (Carpenter and Just, 1989; Just and Carpenter, 1992). Participants were required to read aloud a set of sentences. After each set, participants reported back the final word of each sentence of the most recent set. After every three sets, the number of sentences within a set was increased by one until participants failed to recall one or more words on at least two out of three sets. Participants' reading span score was defined by the number of sentences of the last set in which all final words were recalled correctly. Half a point was subtracted when only one out of three sets was completed correctly. For example, when a participant correctly completed all three two-sentence sets and only one three-sentence set, the test was terminated and a score of 2.5 was obtained. Correctly completing two four-sentence sets and no five-sentence sets resulted in a score of 4. The test started with two practice sets of two sentences and ended with a maximum of five sentences per set, resulting in a reading span score between 1.5 and 5.

### General reading comprehension

Grades 3 and 4 versions of the standardized CITO Reading Comprehension Test (Institute for Educational Measurement; 2010) were used to measure children's reading comprehension skills. This test is part of the standard Dutch pupil monitoring system and is designed to determine general reading comprehension level in primary school children. It contains two modules, each consisting of a text and 25 multiple-choice questions. The questions were designed to tap both the text-base and situation model representation that can be constructed from the text (e.g., Kintsch, 1988) and pertained to either the word, sentence, or text level. Normed proficiency scores were obtained by rescaling students' raw test scores on the 50 items. The rescaling procedure enabled us to compare the results between children from different grades. For Grades 3 and 4, normed proficiency scores could be obtained ranging from −76 to 121. The internal consistency coefficient of the tests was high, with Cronbach's alpha's not less than 0.85 (Feenstra et al., 2010).

## Procedure

Children's legal guardians provided written informed consent based on printed information about the purpose of the study. Participation was voluntary and children received a small gift after the experiment. Like the standard semiannual administration of the CITO reading comprehension test, the CPT was administered in a regular test-taking configuration (i.e., their tables were arranged so there was more room between them and children were facing the front of the class). This is a regular classroom setting in the Netherlands for taking tests such as CITO and was therefore used for administering the CPT. All children were tested in their own classroom. Children received a booklet containing the stories and test statements. They were instructed to read the stories at their own pace. After a story was read, children turned the page to answer the test-statements. They were not allowed to look back. Children got 45 min to complete the booklet. The reading span test was administered individually in a quiet room at school. Children sat at a computer and completed the reading span test at their own pace, which took about 10–20 min, depending on the number of trials completed. Approximately half of the children completed the reading span test before the CPT, whereas the other half completed the reading span test after the CPT. Children never completed both tests on the same day. Because the CITO reading comprehension test had already been administered by school teachers as part of the school's regular assessment program, reading comprehension scores were retrieved from school administrations.

## Statistical analyses

Path analyses using LISREL 9.20 were performed to examine whether the hypothesized model fitted the data and to further explore direct and indirect effects of CPT components and working memory on general reading comprehension. The overall model fit was assessed by chi-square (χ^2^), root mean square error of approximation (RMSEA), and comparative fit index (CFI). A non-significant chi-square statistic, a RMSEA < 0.05, and CFI >0.95 together indicate a strong fit of the data with the model, whereas a RMSEA < 0.08 and a CFI >0.90 indicate an adequate fit (Hu and Bentler, 1999; Kline, 2005). The two path models, as depicted in Figures 1A,B are nested and can be compared statistically.

## Results

### Descriptives statistics

Table 1 shows means, standard deviations, and minima and maxima for each measure. Mean scores on the standardized test for reading comprehension indicate overall average performance. Table 2 shows that all variables are significantly correlated. As found by previous studies (e.g., Potts and Peterson, 1985; Hannon and Daneman, 2001), the TM component is strongly correlated with TI and KI, whereas these latter two are only moderately correlated to each other (*r* = 0.37). KI is strongly correlated with reading comprehension (*r* = 0.48), whereas the other components are moderately correlated with reading comprehension. The working memory measure (i.e., sentence span task) appears to be weakly correlated to all other measures.

**Table 1:** **Means and standard deviations for the component processes task, reading comprehension, and working memory**.

	**Accuracy**		
**Measure**	***M***	***SD***	**Range**
**COMPONENT PROCESSES TASK**^a^			
Text memory (TM)	71.9	15.2	26.7–100
Text inferencing (TI)	70.6	20.9	16.7–100
Knowledge integration (KI)	56.5	19.9	14.3–100
**ADDITIONAL MEASURES**			
Reading comprehension	20.6	17.34	−16–58
Sentence span	2.3	0.50	1.5–3.5

a *Means, standard deviations, and ranges for the components are reported as percentages*.

**Table 2:** **Correlations among CPT components and additional measures**.

**Variable**	**1**	**2**	**3**	**4**	**5**
1. Text memory	–	0.52^***^	0.55^**^	0.38^***^	0.28^***^
2. Text inferencing		–	0.37^**^	0.32^***^	0.16^*^
3. Knowledge integration			–	0.48^***^	0.20^**^
4. Reading comprehension				–	0.24^**^
5. Sentence span					–

* p<0.05;

** p<0.01;

*** *p<0.001*.

### Path analysis

**Figure 2:**
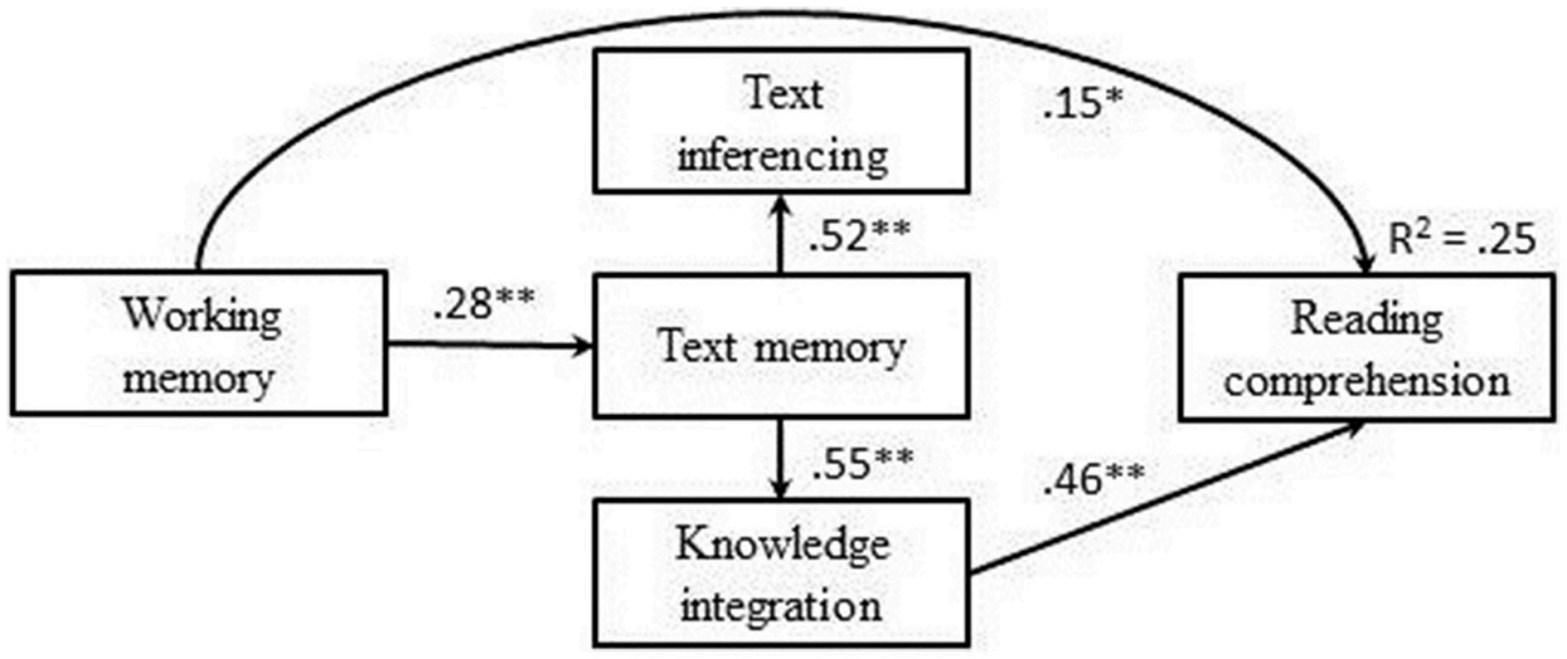
Final model, including the standardized estimates of the variables influencing reading comprehension performance, the significant pathways are indicated with an asterisk, ^*^p < 0.05, ^**^p < 0.01.

First, the full model including both direct and indirect effects, as depicted in Figure 1B, was tested. Together, the fit indices indicated a reasonable model fit. Although the non-significant chi-square, χ^2^(1) = 0.26, *p* = 0.104, and a CFI of 0.99 indicated good fit, the RMSEA of 0.10 indicated a moderate fit, and five paths were not statistically significant: working memory—TI (β = 0.01, *p* = 0.873), working memory—KI (β = 0.05, *p* = 0.489), TM—reading comprehension (β = 0.09, *p* = 0.318), TI—reading comprehension (β = 0.11, *p* = 0.134), and working memory—reading comprehension (β = 0.12, *p* = 0.070). For reasons of parsimony, we removed these paths one by one, starting from the path with the smallest standardized loading. For the first four paths, all Δχ^2^'s were non-significant indicating that the model fit did not worsen. After removing the first four paths, however, the remaining path from working memory to reading comprehension appeared significant (β = 0.15, *p* = 0.026) and was, therefore, not removed from the model; removing this path significantly worsened the model fit (Δχ^2^ = 4.84, *p* = 0.028). Figure 2 shows the results of the final model.

The final model fitted the data well; χ^2^(5) = 8.40, *p* = 0.135, CFI = 0.98, RMSEA = 0.06, explaining 25% (*R*^2^ = 0.25) of variance of reading comprehension. The final model confirmed the hypothesized model (Figure 1A), indicating that indirect effects of working memory on TI and KI were fully mediated by TM, and importantly, KI was the only CPT component predicting unique variance of reading comprehension. As hypothesized, TI did not explain any variance in reading comprehension.

## Discussion

The present study sought to demonstrate that the TI component, as assessed by the CPT, assesses a cognitive skill that does not necessarily contribute to reading comprehension. To our knowledge, this is the first study to include the individual components of the CPT in a single path model and investigate the relative contributions of the CPT components and working memory to general reading comprehension performance in primary school children. In general, our results are consistent with previous findings from CPT studies focusing on adult readers (Hannon and Daneman, 2001; Hannon, 2012), second language learning children (August et al., 2006; Francis et al., 2006), and preschoolers (Hannon and Frias, 2012). We replicated the patterns of correlations among the three CPT components, working memory, and general reading comprehension (Potts and Peterson, 1985; Hannon and Daneman, 2001), indicating that the CPT used in the present study is valid and suitable for primary school children. Furthermore, our hypothesized model explained the present data best, from which we draw several conclusions.

The first important finding is that, according to the present model, the TI component does not contribute to general reading comprehension performance. This is consistent with results reported in previous studies showing that the TI component does not account for any unique variance in general reading comprehension over and above TM (Hannon and Daneman, 2001; Hannon and Frias, 2012). The current study, however, is the first to have directly demonstrated this within a single model. The absence of a path from TI to reading comprehension, provides converging evidence for our argument that the TI component, as assessed by the CPT, does not seem to capture the type of inferential processing that is associated with reading comprehension. The direct path from TM to TI, combined with the absence of a direct path from working memory to TI, indicates that readers depended on memorized text information, rather than on other meaning-based construction processes, for answering TI statements. If readers, irrespective of their working memory capacity in general, fail to memorize explicit text information (TM), they are not able to answer TI statements correctly. Presumably, using the memorized text information, readers may be required to rely on processes like logical reasoning for answering the TI statements (Nunes et al., 2007). These reasoning skills go beyond a typical reading comprehension task and thus the CPT may not reflect real reading comprehension processes. More specifically, the fact that transitive inferences (i.e., A > B, B > C, so A > C) are required in the CPT potentially confounds the interpretations that can be derived from it regarding one's actual reading comprehension performance. This would be in accordance with the strong correlation between the TI component and performance on reasoning tasks found for the original Potts and Peterson paradigm (Hannon and Daneman, 2001), and the finding that logical reasoning skill is relatively independent of working memory (Nunes et al., 2007). Additional research is needed, however, to verify our explanation. This research should at least include readers' logical reasoning skills as a factor in the model or control for these skills when investigating the CPT.

Importantly, by using more narrative-like texts, we excluded the possibility that these results were due to using texts that did not require readers to construct a mental representation during reading. Although narratives were purposefully developed to encourage readers to construct a rich and coherent mental representation, the TI test statements still did not seem to reflect this. Therefore, to enable TI test statements to assess the constructive memory processes (semantic integration and text-based inference) that are not necessarily dependent on the availability of general knowledge, it may be useful to include situational aspects from the narrative in the TI test statements. For example, after reading “the car crashed into the bus” and “the bus was near the crossroads,” it is possible to infer that “the car was near the crossroads.” To do this, the reader does not need specific prior knowledge but is required to construct a coherent perceptual representation of the described story situation (Oakhill, 1982). Although the present study provides suggestions for improvements on the CPT, future research is needed to investigate how the TI test statements can effectively assess text-inferencing processes that are important for the construction of a rich and coherent mental representation of the described situation.

Another important finding is that, according to the final model, the KI component is the only predictor of reading comprehension. This is in accordance with previous research showing that KI was the most important component in explaining variance in both adults' (Hannon and Daneman, 2001) and preschoolers' (Hannon and Frias, 2012) comprehension skills, and was a good predictor of readers' ability to generate bridging inferences (Singer and Ritchot, 1996). The model suggests that TM only indirectly contributes to reading comprehension, through the KI component. The direct path of KI to reading comprehension, combined with the absence of direct paths from TI and TM to reading comprehension, seems to suggest that the KI component is the only component that assesses processes important for comprehension of text. Related to this, the indirect path from working memory to KI, through TM, indicates that when answering KI statements, readers depend on their memory for literal text information as captured by the TM component, rather than on other meaning-based construction processes. If the reader fails to keep explicit information active during reading, they are not able to correctly answer the KI statements.

Finally, after taking into account all the variance of reading comprehension that is explained by the CPT, working memory still accounts for a significant amount of variance in reading comprehension. Consistent with Hannon's (2012) findings, a model including both direct and indirect paths from working memory to reading comprehension fitted the data significantly better than a model only containing indirect paths. This seems to be the case for both children (present study) and adult readers (Hannon, 2012), even though it has been shown that working memory is particularly important to young children's language comprehension (Hannon and Frias, 2012). Our findings suggest that the CPT does not capture all aspects of working memory that are required for reading comprehension. A future version of the CPT may benefit from components tapping into the essence of the story, rather than being highly dependent on text memory. It should provide coverage of comprehension-related inference and integration processes that are directly related to working memory.

Another related direction for future research would be to explore the contribution of other executive functions such as cognitive flexibility and inhibition to reading comprehension, and the present model in particular, because they appear to play a unique role in the development of reading comprehension beyond working memory (e.g., Cartwright, 2012; Guajardo and Cartwright, 2016). For example, to process text effectively readers must actively switch between their own knowledge and text information and integrate both. Therefore, cognitive flexibility possibly explains the relation between knowledge integration and reading comprehension (García-Madruga et al., 2013). This would have implications for intervention, suggesting that children with comprehension difficulties would benefit from promoting the application of WM's executive functioning processes.

To conclude, the present study provides further insight into the CPT's potential as an assessment of individual differences in component processes of reading comprehension. It seems that the TI component, in its current form, assesses inferential processes that are not directly related to reading comprehension. It is, therefore, important that future research further investigates this component and potentially adapts or refines the test statements to make the CPT a more efficient measure of reading comprehension components. The present study contributes to the development of the CPT with respect to both its content and its suitability for assessing primary school children. It is of practical interest that performance on a standardized reading comprehension test for children consists of separate sources of individual differences. Educators need to be aware that the use of a single test of comprehension may not be adequate to assess a child's specific educational needs. It is important to acknowledge that the CPT provides important advantages given its potential to explain individual differences in underlying component processes, compared to currently used general reading comprehension assessments. Detecting reading comprehension difficulties at an early stage and locating its exact sources is essential for providing children with tailored instruction and training.

## Author contributions

SW project design, material development, data collection, data analysis, writing the manuscript. BdK data analysis, writing the manuscript. MdV project design, material development. MvdS project design, material development, reviewing the manuscript.

### Conflict of interest statement

The authors declare that the research was conducted in the absence of any commercial or financial relationships that could be construed as a potential conflict of interest.

## Footnotes

^1^In the present article, “component processes task” (CPT) refers to all tasks based on the Potts and Peterson (1985) paradigm that are used to differentiate between cognitive component processes underlying reading comprehension, including for example the Diagnostic Assessment of Reading Comprehension (DARC; August et al., 2006).
