# Supplementary material for: Does the Component Processes Task Assess Text-Based Inferences Important for Reading Comprehension? A Path Analysis in Primary School Children
Source: Front Psychol. 2016 Jun 14;7:895. doi: 10.3389/fpsyg.2016.00895 (PMC4906010; doi:10.3389/fpsyg.2016.00895)
Supplement: Supplementary file 1 [file DataSheet1.docx]

**Appendix**

**Example Story text**

Dennis loves riding and driving. He has a beautiful bicycle. Every day Dennis rides his bicycle to visits his friends. He also rides a mipper. Mippers are like bicycles, but smaller.

Last year Dennis drove a car for the first time. Dennis drove a car to visit his grandparents. For a moment he did not pay attention and he did not see the other car coming. A loud bang could be heard from afar. The man in the other car got out. He tried to remain calm, but his face turned red. He started to scream and Dennis apologized. Luckily, nobody got hurt.

Recently, Dennis got a plort for his birthday. Plorts are like cars, but bigger. Now Dennis and his grandparents are happy, because they can travel to many places in the plort.

A few days ago Dennis saw a vasker. Vaskers are like plorts, but bigger. Dennis thinks that vaskers are the best. Now he wants to work hard to buy a vasker. Then, he will travel with his grandparents and all his friends together in his new vasker.

**Five-term linear ordering (size)**

Mipper < bicycle < car < plort < vasker

**Test statements**

Text memory:

- Dennis has a bicycle
- Mippers are like bicycles
- Vaskers are bigger than plorts
- Dennis got a plort for Christmas

Text inferencing:

- Vaskers are smaller than cars
- Vaskers are like cars
- Vaskers are bigger than cars

Knowledge integration:

- Vaskers are slower than mippers
- Mippers have two wheels
- Vaskers are bigger than mippers
- Plorts are smaller than mippers
